# Critical evaluation of asthma biomarkers in clinical practice

**DOI:** 10.3389/fmed.2022.969243

**Published:** 2022-10-10

**Authors:** Giuseppe Guida, Diego Bagnasco, Vitina Carriero, Francesca Bertolini, Fabio Luigi Massimo Ricciardolo, Stefania Nicola, Luisa Brussino, Emanuele Nappi, Giovanni Paoletti, Giorgio Walter Canonica, Enrico Heffler

**Affiliations:** ^1^Severe Asthma and Rare Lung Disease Unit, Department of Clinical and Biological Sciences, San Luigi Gonzaga University Hospital, University of Torino, Turin, Italy; ^2^Allergy and Respiratory Diseases, IRCCS Policlinico San Martino, Department of Internal Medicine (DIMI), University of Genoa, Genoa, Italy; ^3^Allergy and Immunology, AO Mauriziano Hospital, University of Turin, Turin, Italy; ^4^IRCCS Humanitas Research Hospital, Milan, Italy; ^5^Department of Biomedical Sciences, Humanitas University, Milan, Italy

**Keywords:** asthma, biomarkers, airway inflammation, eosinophils, exhaled nitric oxide (FENO), sputum, severe asthma, biologics

## Abstract

The advent of personalized medicine has revolutionized the whole approach to the management of asthma, representing the essential basis for future developments. The cornerstones of personalized medicine are the highest precision in diagnosis, individualized prediction of disease evolution, and patient-tailored treatment. To this aim, enormous efforts have been established to discover biomarkers able to predict patients' phenotypes according to clinical, functional, and bio-humoral traits. Biomarkers are objectively measured characteristics used as indicators of biological or pathogenic processes or clinical responses to specific therapeutic interventions. The diagnosis of type-2 asthma, prediction of response to type-2 targeted treatments, and evaluation of the risk of exacerbation and lung function impairment have been associated with biomarkers detectable either in peripheral blood or in airway samples. The surrogate nature of serum biomarkers, set up to be less invasive than sputum analysis or bronchial biopsies, has shown several limits concerning their clinical applicability. Routinely used biomarkers, like peripheral eosinophilia, total IgE, or exhaled nitric oxide, result, even when combined, to be not completely satisfactory in segregating different type-2 asthma phenotypes, particularly in the context of severe asthma where the choice among different biologics is compelling. Moreover, the type-2 low fraction of patients is not only an orphan of biological treatments but is at risk of being misdiagnosed due to the low negative predictive value of type-2 high biomarkers. Sputum inflammatory cell analysis, considered the highest specific biomarker in discriminating eosinophilic inflammation in asthma, and therefore elected as the gold standard in clinical trials and research models, demonstrated many limits in clinical applicability. Many factors may influence the measure of these biomarkers, such as corticosteroid intake, comorbidities, and environmental exposures or habits. Not least, biomarkers variability over time is a confounding factor leading to wrong clinical choices. In this narrative review, we try to explore many aspects concerning the role of routinely used biomarkers in asthma, applying a critical view over the “state of the art” and contemporarily offering an overview of the most recent evidence in this field.

## Introduction

### Heterogeneity of asthma

Asthma has a high prevalence worldwide; it affects patients of all ages and is characterized by a long-lasting impact on patients' and families' life. The burden of the disease in terms of years of life lived with a disability is very high and encompasses the use of medicines, the frequency of hospital admissions, and the costs due to consumable healthcare services (1). The heterogeneity of asthma in terms of onset, natural course, and response to treatment is one of the most outstanding obstacles to the development of efficient strategies to reduce the global asthma burden. On the other hand, the possibility of differentiating underlying pathophysiological mechanisms and grouping patients within disease subtypes (phenotypes) has emerged as the main road evolving from a “blockbuster approach” to a personalized medicine approach (2). The clues of asthma heterogeneity are that similar clinical symptoms that include shortness of breath, wheezing, and cough may be caused by distinct biologic inflammatory pathways (endotypes) that may be unique or more commonly shared among patients presenting with different clinical and functional phenotypes. In this context, the need for asthma biomarkers to identify clinically relevant asthma phenotypes, optimize diagnosis, and guide treatment impetuously emerged (3). However, the scientific community is faced with limits in the use of asthma biomarkers, which are very difficult to overcome. First, the phenotype clustering of patients is very heterogeneous and significantly overlapping, and the methodology used and cohorts examined vary widely (4). Second, currently available biomarkers for clinical practice often overlap within the phenotypes, therefore losing their ability to distinguish clinical and prognostic characteristics. Third, the clinical use of biomarkers in bronchial lavage, bronchial biopsies, and sputum is limited due to invasiveness. In contrast, less invasive biomarkers, such as blood eosinophilia (B-EOS), exhaled nitric oxide (FENO), or total IgE, often lack specificity, regardless of the prespecified threshold used (5). In this narrative review, we try to highlight both the consolidated knowledge and the most controversial aspects of the clinical application of systemic and local biomarkers in asthma with the aim to answer at least some of the practical questions in asthma management. For each biomarker have been reported the best exhaustive references concerning cut-off values, the predictive role of pathophysiological aspects, disease status, and treatment management (6).

### Asthma classification

Asthma clinical presentation is also very heterogeneous. The natural history of the disease can vary from the onset in childhood, often associated with atopy, and clinically fluctuating lifelong with highly variable airflow obstruction, to late-onset forms developing rapidly fixed airway obstruction. The persistence of asthma depends on the interaction, the magnitude, and the timing of exposure, to genetic and environmental factors (7). These mechanisms lead to periods of symptom breakthroughs, suddenly developing into exacerbations. Asthma is therefore classified by most guidelines, according to a “step care” approach to treatment, with the aim of achieving daily asthma control (actual risk) and preventing exacerbations (future risk) by using the lowest level of medication needed (8). Disease control should be sought through the assessment of flare-ups, systemic corticosteroid use in cycles or chronically throughout the year, respiratory function parameters, and symptoms score as evaluated through the asthma control test (ACT).

### Definition of severe asthma

Although severe asthma is a well-known disease subtype, there is no single definition of this condition (9). In fact, there are different definitions that consider factors such as the number of flare-ups, hospitalizations, disease control, and systemic corticosteroid use. The most frequently used definition is that of the 2014 ATS/ERS guidelines (10), also adopted by the latest GINA report (11) in which a patient with asthma is defined as severe if, despite treatment with inhaled drugs at step 4-5 of the GINA document, ensuring that there is good adherence to prescribed therapies and treatment of comorbidities, he or she is uncontrolled. Although the differences between various severe asthma definitions are small, they may lead to an uneven classification of these patients. One of the first steps in the path of defining a patient as a severe asthmatic is the assessment of therapy adherence. The use of the correct therapy, at the prescribed dosage, and with an appropriate inhalator technique is fundamental as it allows the distinction between patients with severe asthma and patients with uncontrolled asthma. In fact, a patient with uncontrolled asthma, not due to the disease itself, but due to poor adherence to the prescribed medications, cannot be considered a severe asthmatic (11). Therefore, once an asthma diagnosis has been confirmed, we need to take a careful pharmacological history in order to evaluate the patient's tendency to take prescribed medications, and only after having ascertained the correct intake, we can consider the patient as a severe asthmatic. Of critical importance, even in the definition of uncontrolled severe asthma, is the search for comorbidities. Starting from the definition of severe asthma, the evaluation of other pathologies that can make the control of the disease more complex is crucial. The main comorbidities linked to asthma are chronic rhinosinusitis, gastroesophageal reflux disease, allergic diseases, and bronchiectasis (12). The poor control of these pathologies negatively impacts asthma control itself.

### Phenotype and endotype

Patients with severe asthma frequently require systemic corticosteroid therapy and are notoriously burdened by both short and long-term side effects (13). Highly effective biological drugs for severe asthma are available and have a better safety profile than systemic corticosteroids (13). Since the early 2000s, the idea of phenotyping (14, 15) has been developed with the aim to improve treatment outcomes and correctly framing patients. Consequently, distinct asthma endotypes, namely pathophysiological mechanisms underlining the pathology, were also identified. In the case of severe asthma patients, the first distinction is between type two (T2) and non-T2 (16) inflammation. Most patients have T2 inflammation, which is generally responsive to steroid therapies, and whose biological mechanisms are driven by the inflammatory cascade produced by type two T helper cells (TH2) (17) and type 2 innate lymphoid cells (ILC2). The inflammatory cytokines involved in the T2 type of asthma are mainly interleukin (IL) 5, 4, and 13, accompanied by those defined as alarmins, respectively IL-33, IL-25, and thymic stromal lymphopoietin (TSLP) (18). Associated with these cytokines, there are also immunoglobulins type E (IgE), produced by B cells. In contrast, the non-T2 form of inflammation involves other cells, such as neutrophils, and other proinflammatory cytokines such as IL-17, IL22, IFN γ, and from other cells such as TH1 and TH17 (19). This type of inflammation, which is still poorly understood, causes forms of asthma that are quite complicated to treat with the drugs available at this time. Regarding asthma phenotypes, in addition to the distinction between T2 and non-T2 inflammation, patients can also be subdivided into allergic and non-allergic and into early-onset and late-onset (4). In daily clinical practice, the importance of phenotyping our patients is linked to the possibility of a precise recognition of the mechanisms underlying its disease and choosing the most precise therapy, an approach defined as Precision medicine (20).

### Role of biomarkers in precision medicine

Biomarkers are directly associated with the concept of phenotyping and endotyping in asthma. The objective is to try, through the presence of several markers, whether clinical, systemic, or local, to predict the response to a given therapy (21, 22). In the context of severe asthma, the main biomarkers that have been developed are B-EOS, IgE, FENO, and the inflammatory sputum cell analysis, all of which will be discussed below.

## Systemic biomarkers

### Blood eosinophils

Eosinophils and their secretory mediators play an essential role in the pathogenesis of many inflammatory disorders. Eosinophils develop and differentiate in bone marrow in response to infective, inflammatory, and allergic stimulation, driven by cytokine IL-5. Eosinophil-related diseases are characterized by evidence of increased blood and/or tissue eosinophils with or without evidence of their activation. Eosinophils are recruited into tissues in response to chemokines, mainly of the eotaxin family, and are present in the blood for only a few hours, while they survive in tissues for several weeks, where they activate, cooperate with inflammatory tissue damage, and reside further due to delayed apoptosis (23).

### Eosinophils and pathophysiology of asthma

The role of eosinophils in asthma inflammation is well known as they mediate asthma development and airway remodeling (24, 25). Eosinophils release several specific mediators able to promote bronchial inflammation. Among them, are the major basic protein (MBP), eosinophil cationic protein (ECP), and lipid mediators such as cysteinyl leukotrienes (cysLTs) (26). Recent observations, both in animal models and in human samples, have shown the existence of distinct eosinophil subtypes, distinguishable in inflammatory (iEOS) and resident (rEOS). rEOS, also described as homeostatic eosinophils, are ring shaped-nucleus cells, mainly detected in the lung parenchyma, and regulate homeostatic processes, host defense as well as negatively Th2 cell responses at a steady state. Their trafficking to the lungs is guided by eotaxin-1 and is IL-5 independent and they are phenotypically characterized by the expression of intermediate levels of Siglec-F and CD125. On the other hand, iEOS, defined as Siglec-F^hi^CD125^int^CD62L^−^CD101^hi^ cells with a segmented nucleus, are IL-5-dependent cells localized in the peribronchial areas and able to be recruited to the sites of Th2 inflammatory responses (27). The difference between these two cell types may lead to new hypotheses about the role of eosinophils and the need to analyze their function better.

### Blood eosinophils cut-offs

Blood eosinophilia levels vary in different eosinophil-related diseases, with higher levels in systemic diseases to lower levels in localized ones. Under inflammatory conditions, eosinophil numbers expand greatly, as in the case of allergen airway provocation that can result in B-EOS within hours of the challenge. Cut-offs of total eosinophil count in the blood can be helpful in the initial workup for patients presenting with eosinophilia or hypereosinophilia. B-EOS values below 500 cells/μL can be found in allergic rhinitis and asthma, although sometimes rising to the levels of 1,500 cells/μL, overlapping with that of other diseases characterized by higher B-EOS values, such as eosinophilic granulomatosis with polyangiitis (EGPA) or allergic bronchopulmonary aspergillosis (ABPA), or diseases with a prominent eosinophilic infiltration, such as chronic rhinosinusitis with nasal polyps (CRSwNP), and cutaneous drug-induced allergy. Extremely high total eosinophil counts (e.g., >5,000 cells/μL) should also rise the suspicion of hypereosinophilic syndrome (HES) and its myeloid variants (28).

### Blood eosinophils as biomarkers of airway eosinophilia

Blood eosinophils are considered a suitable biomarker as their measurement is inexpensive and widely available. Asthmatic patients often have normal B-EOS values, although higher B-EOS have been shown to correlate directly with symptom scores and inversely with FEV1 in both children and adults, independently of atopy (29). Whether B-EOS may be considered a good surrogate marker of airway eosinophilia is highly debated. A moderate-to-good correlation of B-EOS with sputum eosinophils (S-EOS) in large cohorts of asthmatics has been reported. In a cohort of unselected patients with asthma (*n* = 508), a significant positive relationship between B-EOS count and percentage of S-EOS count was reported and B-EOS count >220/mm^3^ resulted in good predictors of S-EOS ≥ 3% with a 77% sensitivity and 70% specificity (30). The validation of B-EOS to detect eosinophilic airway inflammation has been shown comparing two independent cohorts, mild-to-moderate asthma vs. moderate-to-severe asthma. A cut-off point of ≥0.27 × 10^9^/L B-EOS was able to differentiate eosinophilic inflammation of ≥3% with a sensitivity of 78% and specificity of 91% (31). Other studies, yet concluded that, despite being statistically associated, the predictor power of B-EOS for S-EOS was poor. Accordingly, within the SARP population, the sensitivity and specificity of B-EOS counts >300/μL to detect an “eosinophil phenotype” based on S-EOS counts >2% were 59 and 65%, respectively. The mathematical consequence is that a B-EOS count of < 300/μL yields a 41% false-negative and likewise, a false-positive rate of 35% would misclassify patients with an S-EOS count of < 2%. The attempt to restrict the observation to subjects with severe asthma only and to rise the cut-off of S-EOS counts to more than 3% failed (32). Moderately better results were demonstrated in a more restricted population of 75 uncontrolled asthmatic patients, yielding a significant positive relationship between the percentage of S-EOS and B-EOS (*r* = 0.3647), yet limited by the use of the cut-off point of B-EOS of 1.5% of WBC (33). Higher sensitivity and specificity and AUC as a biomarker of S-EOS (≥3%) in a population of uncontrolled asthmatics were reported to increase the peripheral B-EOS cut-off percentage to 2.7% (34). A systematic review and meta-analysis including 14 studies investigating B-EOS as a predictor marker for airway eosinophilia in patients with asthma yielded overall a modest capacity to distinguish between patients with or without airway eosinophilia (AUC of 0.78) and either eosinophil ≥2 or >3% did not affect the accuracy of the test (35). The diagnostic accuracy of B-EOS to detect S-EOS did not significantly differ between obese and nonobese, atopic and nonatopic, (ex-)smoking and never-smoking, and severe and mild-to-moderate asthma patients (36). Table 1 summarizes the cut-off and predicting values of B-EOS for airway eosinophilic inflammation. Overall, B-EOS remains an appropriate asthma biomarker with higher feasibility. The conflicting evidence of correspondence between B-EOS and S-EOS values highlights the need for an accurate cut-off determination.

**Table 1:** Blood eosinophils cut-off as predictor of airways eosinophilia.

**Asthma populations**	**B-EOS cut-off (μl)**	**S-EOS cut-off (%)**	**Sensitivity (%)**	**Specificity (%)**
Unselected (30)	>220	≥3%	77	70
Mild-to-severe asthma (31)	>270	≥3%	78	91
Mild to severe (SARP) (32)	>300	>2%	59	65
Uncontrolled asthmatics (33)	1.5% of WBC	≥ 2.5%	61.5	78.3
Uncontrolled asthmatics (34)	2.7% of WBC	≥3%	92.2	75.8
Poorly controlled with high-dose ICS (Reslizumab trial) (37)	>400	>3%		
Severe eosinophilic asthma (DREAM trial)	≥300	≥3%		
Severe eosinophilic asthma (MENSA, MUSCA trials)	>150	-		
Severe uncontrolled asthma (38)	>300 (ELEN Index*)	≥2%		
Severe eosinophilic asthma (SIROCCO,CALIMA trials)	≥300	-		

*Mathematically weighted ratios of three blood cell populations, Eosinophils/Lymphocytes and Eosinophils/Neutrophils (ELEN), as predictor variables of sputum eosinophilia.

### Blood eosinophils as a biomarker of biologic treatment in severe asthma

In the clinical practice setting, for several years already, the role of eosinophils has become increasingly central as a biomarker for predicting response to therapy. Indeed, the first mepolizumab (anti-IL-5 mAb) studies, showed that patients with higher eosinophilia responded better to therapy, compared to a more heterogeneous sample, which had not been previously selected according to the blood values of these cells (39, 40). Then over time, it was seen that the presence of high B-EOS values was an important biomarker of therapeutic response. The appropriate cut-off of B-EOS to predict airway eosinophilia in severe asthma has given conflicting results. In a population selected for treatment with reslizumab, another anti-IL-5 mAb, B-EOS counts of >400/μL may be able to improve the prediction of S-EOS counts of >3% (37). The DREAM study identified a B-EOS count of 300/μL or greater as a high predictive biomarker of response to mepolizumab (41), but the subsequent clinical trials enrolled severe asthmatic patients with a peripheral eosinophil count of at least 150 cells/μL at screening or at least 300 cells /μL at some time during the previous year (42, 43). In studies in real life, concerning the efficacy of anti-IL-5 drugs, regardless of the number of eosinophils, provided that above 300 cells/μl, B-EOS correlated with risk of asthma exacerbations, the decline in respiratory function, mortality, and systemic corticosteroid dependence (44). Moreover, a relationship between the reduction of eosinophil, with anti-IL-5 drugs, and the consequent decrease in the frequency of exacerbations was reported (45). As for asthma, also other comorbidities related to the T2 inflammation, are relatable to the increase in the number of B-EOS, particularly CRSwNP and atopic dermatitis (46). This suggests that B-EOS, despite remaining a good predictive biomarker, can provide a more precise prediction of asthma treatment response if associated with other biomarkers.

Benralizumab, the anti-IL-5 receptor humanized IgG1κ monoclonal antibody, demonstrated a dramatic effect on B-EOS count in Phase I trials, reaching eosinopenia after 8–12 weeks of intravenous doses >0.03 mg/kg (47). The comparison of a single intravenous dose with a subcutaneous strategy caused decreased B-EOS counts by 100% in both arms after 21 days, suggesting suppression of bone marrow eosinophil production (48). The use of B-EOS as a surrogate marker of S-EOS has been applied in subsequent trials. The ELEN index, based on two predictor variables (the ratio of blood eosinophils to lymphocytes, and the ratio of blood eosinophils to neutrophils) was used to classify participants as having either < 2% or at least 2% S-EOS, without the need to collect sputum in a phase 2b randomized dose-ranging study enrolling uncontrolled eosinophilic asthma. This study found that in the group with baseline B-EOS cut-off of at least 300 cells/μL, significantly lower exacerbation rates in the benralizumab 20mg groups were observed (38). The consequence has been the application of B-EOS cut-offs of ≥300 eosinophils/μL, to define severe uncontrolled eosinophilic asthma in 3 phases by evaluating the efficacy and safety of benralizumab treatment (49, 50). Of note, a decrease in the rate of exacerbations was observed even in the groups with baseline eosinophil count < 300/μL relative to placebo. A *post hoc* subanalysis of both trials compared the efficacy and safety of benralizumab in patients with eosinophil cut-off count of either ≥ 150 or < 150 cells/μL, concluding that benralizumab was effective in patients with asthma and blood B-EOS ≥150 cells/μl (51). The combination of B-EOS basal threshold with clinical traits can help in predicting the response to benralizumab, therefore in the presence of OCS use, CRSwNP, prebronchodilator FVC < 65% of predicted, the response rate was high also in < 300 cells/μL baseline group (52).

### Variability of blood eosinophilia

The cross-sectional nature of many study populations represents one of the most evident limits in the role of blood eosinophilia as a biomarker. Actually, significant variability of B-EOS count in the same patient over time and according to treatment status has to be considered. Repeated B-EOS measurements during a five-year follow-up of severe asthmatics showed a high probability to cross the count above or below the thresholds of 150, 300, 400, and 500 cells/μL. When the 300 cells/mL threshold was chosen only 22% of the participants continued to exceed the cut-off value over time (34).

### IgE and asthma

As eosinophils, also IgE is a product of the T2 inflammation pathway. IgE levels are usually associated with specific sensitization to several allergens, both seasonal and perennial, but also with inflammatory, immunologic or hematologic disorders (53). IgE levels are important both total and specific one, particularly the second is crucial for the identification of allergen sensitization as possible triggers of asthma. Just IgE was the target of the first monoclonal antibody in severe asthma, Omalizumab, able to bind the C3 region of IgE Fc fragment, determining a reduction of free IgE available to bind their receptors on cells (54). Generally, the clinical utility of measuring total IgE serum levels is limited by its low specificity. Total IgE, contrary to what could be expected, does not consistently discriminate among asthmatic populations between atopic and non-atopic. Among patients from the International Severe Asthma Registry (ISAR) patients with atopy had a median IgE value of 535 KU/L compared to non-atopic 224.3 KU/L (55). Even applying cluster analysis combining clinical physiological and biological traits, total IgE was not able to discriminate among the different groups (56). The sensitivity and specificity of total IgE as a predictor of airway eosinophilia are quite weak and an AUC of 0.62 has been reported. At a sensitivity of ≥ 95% FENO, B-EOS and total IgE had a comparable specificity, but the sensitivity of total IgE was significantly lower compared to the other biomarkers (0.47) (36).

A broader application of total IgE is that of identifying patients with T2 inflammation. Actually, gene expression analyses reported that subjects with Th2-high asthma had higher serum IgE levels than Th2-low (244 IU/ml vs 125 IU/ml)(57). In an unselected population of patients with severe asthma, a cut-off of IgE > 150 IU/mL was chosen as one of the type-2 biomarkers being detectable in 42% of patients (58). The International Severe Asthma Registry (ISAR) applied a lower cut-off of ≥75 kU/L, which yielded a prevalence of 59% of IgE-positive patients. The likelihood of having another T2 positive biomarker was 59 and 65% respectively for B-EOS and FENO. This study reported a cluster of patients, accounting for 6% of the whole population, that is characterized by very high levels of IgE (1,932 kU/L), which is clinically associated with the youngest age, the longest duration of asthma, obesity, and poor lung function (58).

Even if IgE is the biomarker for the eligibility for omalizumab treatment of severe asthmatics, a role as a response predictor to omalizumab in different severe allergic asthma populations was not demonstrated. The response to omalizumab, in terms of ACQ clinical variation, was not associated with IgE levels (59). A Spanish real-life study observed a greater decrease of IgE after omalizumab interruption in the failure group, suggesting that the faster IgE decreases, the earlier asthma relapses (60). Accordingly the Xolair Persistency Of Response After Long-Term Therapy (XPORT) trial showed that discontinuation of omalizumab was associated with a decrease in total IgE levels and an increase in free IgE levels as well as an increase in basophil expression of the high-affinity IgE receptor (61). Dupilumab demonstrated its clinical efficacy in patients with both severe allergic asthma, defined by total serum IgE > 30 IU/mL and > 1 perennial aeroallergen-specific IgE >0.35 kU/L at baseline, and not allergic. A reduction in total serum IgE occurred for both the allergic and non-allergic patients. A baseline total IgE > 700 IU/mL did not influence the rate of adjusted annualized severe exacerbation rate (62). Given these observations, the major clinical applications of this biomarker are to predict the response to anti-IgE therapy and to determine the optimal dosage of omalizumab.

## Local biomarkers

### Sputum inflammatory cells

#### Morphologic sputum cell analysis

Sputum inflammatory cell analysis defines the different inflammatory phenotypes of asthma and is currently the most valid, specific, and noninvasive method for measuring airway inflammation. Processing and analysis are standardized and reliability, validity, and responsiveness are proven (63). Differential sputum cell count is performed after cytospin centrifugation and staining, on a minimum of 400 nonsquamous cells reported as the relative numbers of eosinophils, neutrophils, macrophages, lymphocytes, and bronchial epithelial cells expressed as a percentage of total nonsquamous cells. The normal range of cell counts in induced sputum for nonsmoking healthy adults has not been standardized, yet Spanevello and co-workers reported reference values and the distribution of cells in induced sputum from a population of ninety-six healthy, nonatopic, nonsmoking volunteers without airway hyperreactivity (64) that is comparable to the values from the population in Jose Belda's Study, that even including atopic subjects did not show any difference (65) (Table 2).

**Table 2:** Reference values of sputum inflammatory cells in healthy subjects.

**Cell type**	**Spanevello A, Italy**		**Belda J, Canada**		**Luo W, Guangzhou, China**	
	**Mean (%) ±SD**	**Upper value**	**Mean (%) ±SD**	**Upper value§**	**Mean (%) ±SD**	**Upper value**
TCC	2.7 ± 2.5	14.2	4.1 ± 4.8	9.7	2.1 ± 1.0	4.5 (× 106/g)
Macrophages	69.2 ± 13	95	58.8 ± 21.0	86.1	58.9 ± 17.7	90.0
Neutrophils	27.3 ± 13	49.2	37.5 ± 20.1	64.4	38.3 ± 17.7	72.9
Eosinophils	0.6 ± 0.8	2.4	0.4 ± 0.9	1.1	0.3 (1.1)	2.0
Lymphocytes	1.0 ± 1.2	5	1.0 ± 1.1	2.6	1.0 (1.8)	4.5
Epithelial cells	1.5 ± 1.8	8.2	1.6 ± 3.9	4.4	0.0(1.5)	5.5

TCC, total cell count; §90pertentile.

#### Eosinophilic asthma and sputum cell cut-offs

A plethora of experiments confirmed the key role of airway eosinophils in the pathophysiology of asthma and eosinophilic asthma as being the most common phenotype accounting for ~50–60% of the total asthma population, up to 80% in corticosteroid-naive subjects. The definition of eosinophilic asthma implies that eosinophils are the dominant cells responsible for the pathophysiological changes of the disease. On the other hand, the existence of “eosinophilic asthma” is coupled with the recognition of noneosinophilic asthma (NEA). Sputum cell count identifies eosinophilic, neutrophilic, both eosinophilic and neutrophilic (mixed), and paucigranulocytic types of asthma, yet different cut-off values have been applied for eosinophilic and neutrophilic differential cell count, ranging, respectively, from above 1–3% and above 40–76% (Table 3) (30, 66–69). A study designed to evaluate the reproducibility of an inflammatory subtype with different eosinophil cut points (starting at 1% eosinophils), showed that all cut points >2% were reproducible and a 3% cut point resulted in the highest power to distinguish eosinophilic from non-eosinophilic airway inflammation (70). Eosinophilia may yet result persistently observable under multiple sputum examinations only in 22% of mild to moderate asthma, being more often present on at least 1 occasion (intermittent eosinophilia, 31%) (71). Further studies reported the stability of phenotypes defined by sputum biomarkers to be weak (72).

**Table 3:** Cut-off values for the different induced sputum inflammatory phenotypes.

**References**	**Eosinophilic type**		**Neutrophilic type**		**Mixed type**		**Paucigranulocytic type**	
	**EOS (%)**	**NEU (%)**	**EOS (%)**	**NEU (%)**	**EOS (%)**	**NEU (%)**	**EOS (%)**	**NEU (%)**
Simpson et al. (66)	>1.01	≤ 61	< 1	>61	>1.01	>61	< 1	≤ 61
Hastie et al. (67)	≥2	< 40	< 2	≥40	≥2	≥40	< 2	< 40
Schleich et al. (30) Demarcheeet al. (68)	≥3	< 76	< 3	≥76	≥3	≥76	< 3	< 76
Vijverberg et al. (3) Ntontsi et al. (69) Shi et al. (73)	≥3	< 61	< 3	≥ 61	≥3	≥ 61	< 3	< 61

#### Sputum eosinophils biomarker of allergic asthma and AHR

The finding of activated eosinophils (EG2+) both in bronchoalveolar lavage (BAL) and bronchial biopsies from mild atopic asthmatic patients (74), as well as after allergen challenge (75) demonstrated the central role of eosinophils in the effector phase of the late allergic inflammatory response. The delayed bronchoconstrictor response that occurs 4–6 h after allergen challenge, named the late asthmatic response (LAR), is characterized by prolonged AHR, pronounced airway eosinophilia, and release of eosinophil cationic protein (ECP), markers of eosinophils activation. Airway eosinophils persist in allergic asthmatic far beyond the peak of LAR, lasting even for 7 days after allergen inhalation, being sustained by the action of chemotactic factors (76), and returning to baseline within a few weeks. Initial clinical findings confirmed the detection of eosinophils in induced sputum from allergic asthmatics not only after specific bronchial allergen challenges. Foresi and coauthors reported a median EG2+ sputum eosinophils count of 16.7% in mild perennial asthmatics sensitized to dermatophagoides and showed an inverse correlation between methacholine PD20 and S-EOS (77). On the contrary, a dissociation between airway inflammation and AHR in allergic asthma resulted from a more extended cohort of mild to moderate asthmatics sensitized to perennial allergens (77). In other cohorts of patients with mild atopic intermittent asthma there were no correlations between induced S-EOS and PC20, even comparing recently diagnosed asthma (< 5 years) and longer-standing asthma (>5 years)(78). To weight the effect of eosinophils on AHR, a stepwise multiple regression analysis was performed in a very large group of mild to moderate steroid-naive people with asthma, and a relationship between S-EOS count and AHR to methacholine was found, yet the extent of S-EOS accounting for 16% of the variation in PC20 Methacholine (79). This positive correlation between S-EOS and PC20 methacholine resembles that reported by Jatakanon et al. (80) that also found a correlation for both variables with FENO (Table 4).

**Table 4:** Relationship between sputum eosinophils and AHR in asthmatic populations.

**Asthma population**	**Patient number *(N)*, [REF]**	**Allergen**	**Methacholine Mean PC20 mg/ml**	**Mean (SE) sputum Eos (%)**	**Correlation**
Mild intermittent (recent diagnosis)	*N =* 30, [Boulet LP]	Cat, dog, house-dust mite or cockroach	4.9 (2.3)	0.9 (0.5–2.2)	NO
Mild intermittent (diagnosis > 5 years)	*N =* 30, [Boulet LP]	Cat, dog, house-dust mite or cockroach	2.9 (2.2)	0.9 (0.4–1.9)	NO
Mild persistent Off ICS treatment (6 month)	*N =* 30, [Foresi A]	House-dust mite	0.42 to 2.6 mmol	16.7 ± 5.06	YES *r =*-0.52
Mild to moderate persistent Off ICS treatment (1 month)	*N =* 28 [Crimi E]	House-dust mite or pet dandruff	0.099	8.5± 1.9	NO R = 0.24
Mild to moderate naïve Off ICS treatment (6 weeks)	*N =* 118,[ R. Louis]	House-dust mites, grass, birch, weed, cat and dog dander, mold mixture	1.10 (0.03–16)	10.9 (13.6)	YES *R =*-0.40
Mild to moderate naïve Off ICS treatment (3 months)	*N =* 35 [A Jatakanon]	Non known	0.52 (1.23)	6.8 (1.2)	YES *r =* −0.40

#### Sputum eosinophils biomarker of symptoms and asthma control

The S-EOS count does not differentiate between patients with controlled asthma (*n* = 158) or severe asthma (*n* = 163) with a mean eosinophils % of 4.1 ± 3.1 and 4.4 ± 5, respectively, as reported by the ENFUMOSA cross-sectional observational study (81). Interestingly those with severe disease had a significantly greater number of neutrophils in their sputum. Mild persistent asthmatics not taking ICS (82) were analyzed for discrepancies between clinical, physiological, and inflammatory scores, resulting in the presence of a high S-EOS count correlated to lower asthma control. Yet a high proportion of mild asthmatics had persistent S-EOS despite a good mean clinical and physiological score. In another study mild to moderate steroid-naïve uncontrolled patients did not differ in respiratory symptoms, quality of life, FEV1, and PD20FEV1 whether they had high (>3%) or low S-EOS as well as their response to ICS (83). In a cross-sectional analysis of 995 persistent mild to moderate patients, S-EOS (>2%) was found to correlate with better control and younger age. On the contrary FEV1 < 70% of predicted, daytime symptoms or short-acting beta-agonist use ≥6 days per week, nocturnal awakening ≥2 nights per week was associated with a lower % of eosinophilic asthma. On the other hand, persistent S-EOS is associated with a lower PD20 methacholine response (71). Within the Severe Asthma Research Program (SARP), which included a population of severe and non-severe patients with and without corticosteroid treatment, S-EOS ≥2% was associated with daily use of β-agonist and daily wheeze, while both increased EOS and increased neutrophils (mixed) had lowest lung function measures, worse asthma control, greatest symptoms and use of healthcare resources (67). In the retrospective series of 508 asthmatics by Schleich, independent predictors of S-EOS were B-EOS %, FEV1/FVC, FENO, and IgE levels. Higher atopy, higher AHR, and lower asthma control were associated with the eosinophilic phenotype; the mixed granulocytic phenotype was also explored, being associated with the lowest lung function and the highest degree of AHR (30). The cross-sectional assessment of adults with severe and mild/moderate asthma from the Unbiased Biomarkers for the Prediction of Respiratory Disease Outcomes (U-BIOPRED) project found the higher S-EOS count associated with two severe asthma groups (smokers and non-smokers) compared to the mild/moderate asthma group and negative association between log S-EOS and FEV1. The severe asthma patients were characterized by higher BMI, a median of 2.5 exacerbations in the preceding 12 months, and a higher rate of intensive care unit admissions (84). Regarding atopy and smoking habits, a retrospective cross-sectional study on 833 asthmatics recruited from the University Asthma Clinic of Liege, confirmed that the distribution among the groups was not correlated to the sputum inflammatory phenotype. Taken together all these data suggest that in mild/moderate patients, naïve to steroid treatment, S-EOS partially correlates with the worst control and symptoms, but the paradigm is overturned when patients are ICS treated. In the severe forms of asthma, the persistence of eosinophilia is associated with a lack of treatment response, and worse control, and can evolve into the worst airway obstruction. Yet the cohorts including all degrees of severity and treatment show that S-EOS has not the capacity of segregating patients according to atopy, smoking, and severity of the disease (Table 5).

**Table 5:** Associations between sputum inflammatory phenotypes and clinical outcomes.

**Asthma population**	**Patient number *(N)*, [REF]**	**Sputum cells**	**Clinical marker**
Mild asthma Steroid naive	*N =* 213 [Boulay et al. (82)]	Eos > 2%	↓ control
Mild to moderate steroid naïve nonsmoking	*N =* 51, [Godon et al. (83); 20: 1364–1369]	Eos > 2%	→ Symptoms → QoL → FEV1 → BHR
Mild to moderate Treated ICS/OCS	*N =* 995 [McGrath et al. (71)]	Eos ≥3 %	↑control ↓age ↓BMI ↓BHR
Mild to severe Treated and untreated with ICS nonsmoking (SARP cohort)	*N =* 242 [Hastie et al. (67)]	Eos ≥2%	↑use of β-agonist ↑daily wheeze
		Mixed eos / Neu	↓ FEV1 ↑ Symptoms ↑Healthcare resources
Mild to severe Treated and untreated with ICS Included smokers	*N =* 508 [Schleich et al. (30)]	Eos ≥2%	↑ Atopy ↑ AHR ↓ control (ACT)
		Mixed eos /Neu	↑AHR ↓FEV1
Mild to severe Treated ICS/OCS Smokers included (U-BIOPRED cohort)	*N =* 509 [Shaw et al. (84)]	Eos > 1,9%	↓FEV1 ↑BMI ↑Exhacerbations ↑Intensive care

↓lower; ↑higher; → no correlation; QoL = quality of life; BHR, bronchial hyperresponsiveness.

#### Sputum eosinophils biomarkers of corticosteroid treatment response

Eosinophilic airway inflammation is notoriously steroid-sensitive and ICS is currently the most effective treatment for asthma. Inhaled fluticasone propionate (500 μg twice daily) caused a significant decrease in S-EOS from a mean value of 2.85 to 0.68(%) in a randomized, double-blind, placebo-controlled, parallel study of 25 patients with mild atopic asthma. Cessation of ICS treatment on the other hand worsens S-EOS count (up to 8.14%) (85) and this increase is a predictor of loss of asthma control and risk of exacerbations. The benefits of airway eosinophilic inflammation in mild asthma is obtained also through regularly low-dose inhaled fluticasone during a follow-up period of 1 year of stable, well-controlled asthma. The time to exacerbation tended to be shorter in subjects with an S-EOS count >5% (86). S-EOS was also evaluated and confirmed for predicting the clinical response, as measured by a change in FEV1, PC20 Mch, and asthma QOL in unstable asthmatics treated for 2-week with either OCS or ICS (87). A clinical strategy, based on re-administration of ICS when a change in the.8% S-EOS threshold was reached, lowered the rates of asthma deterioration and the number of individuals treated with ICSs by 48%. In addition, sputum examination can detect an increase in airway eosinophils up to 3 months before the development of a clinical exacerbation (88). Adjustments of therapy with ICS by assessing S-EOS help to maintain adequate asthma control, especially in reducing the risks of asthma exacerbation. This strategy has been elegantly proved by RH. Green who recruited 74 patients with moderate to severe asthma and showed over 12 months in the sputum management group (maintenance of an S-EOS count at below 3%) an S-EOS count lower than 63% than in the BTS management group and significantly fewer severe asthma exacerbations (89). An eosinophil count of >3% represents a cut-point that is both above the normal range and is associated with a favorable response to corticosteroid therapy. According to this observation, the response to ICS stratified by baseline S-EOS count showed that subjects with elevated S-EOS had a significant improvement in lung function with corticosteroids (87). On the other hand, the lack of improvement in subjects with S-EOS of < 3%, raises the question of whether NEA is steroid-resistant. Applying the sputum cell counts to guide treatment in moderate to severe asthma, Jayaram et al. showed that keeping sputum eosinophils < or =2% was associated with a longer time to the first exacerbation and a reduced number of eosinophilic exacerbations. It is of note that sputum guided strategy did not influence the frequency of noneosinophilic exacerbations, which were the most common (90). Overall, the effectiveness of strategies based on sputum examination to guide treatment decisions not only reflects lung function improvement, and a decrease in asthma symptoms and exacerbations but allows to avoid overtreatment in patients who do not have airway eosinophils. The results are more consistent in moderate to severe asthmatics compared to mild selected populations (91). Another consequence of steroid sensitivity of eosinophilic asthma is to argue for either non-adherence or inadequate corticosteroid dosing when the persistence of sputum eosinophilia is detected in mild-moderate asthma (92).

#### The non-eosinophilic phenotype of asthma

One of the first suggestions about the existence of non-eosinophilic asthma is the report from Turner et al. finding normal levels of S-EOS in subjects with symptomatic asthma, frequent use of beta2-agonist, and AHR (93). About one-third of the steroid-naive asthma group are noneosinophilic (79) and monthly repeated sputum induction assessments over 5 months with a cut point of 3% eosinophils should be used to distinguish eosinophilic from non-eosinophilic asthma (70). More recently in a very large cohort of ICS-negative mild to moderate asthmatics, 64% of the subjects had fewer than 2% eosinophils (71). Actually, the population attributable risk of asthma because of eosinophilic inflammation in epidemiologic observations was about 50% (94). Bronchial biopsy studies confirmed that the non-eosinophilic phenotype is present in the airway mucosa, as well as the airway lumen, therefore NEA is not just a phenomenon of sputum. As previously reported, the sum of neutrophilic and paucigranulocytic types of asthma accounted for slightly more than 50% in steroid naïve patients and the ratio did not change within steroid-treated patients (30), as confirmed by other studies including the patient in ICS treatment (66, 69). Pavord and colleagues demonstrated the poor response to a 2 months course of budesonide 400 mcg twice daily of NEA in terms of an increase in PC20 and a decrease in symptoms (95). When ICS treatment was withdrawn in 94 patients with persistent asthma, patients classified as EA resulted not only in taking more often ICS and at higher doses, but in losing control in a higher percentage than NEA. Yet, after recommencing the treatment (fluticasone 1000mcg), the response in terms of ACQ, FEV1, PC20AMP (adenosine monophosphate), and FENO was significantly greater in patients with EA than in those with NEA (96). Finally, another proof of the poor response of NA to corticosteroids is the absence of effect on non-eosinophilic exacerbations in the sputum cell counts guided treatment trial by Deykin et al. (88).

#### The neutrophilic phenotype of asthma

Neutrophilic asthma accounts for 5.4–15.7% in different series unselected for severity or ongoing treatment (30, 69). Older age and functional residual capacity (FRC) were independent factors associated with sputum neutrophilia and patients receiving moderate to high dose ICS had higher sputum neutrophil count than patients receiving low dose ICS. The history of ICS intake for 3–6 months was all associated with neutrophilia (73). Other characteristics associated with neutrophilic asthma are an increased prevalence of fixed airflow obstruction, a lesser prevalence of atopy, and a greater prevalence of past smoking (66). In one cohort, GERD was associated with sputum neutrophilia (97). Lower respiratory tract viral infections have been associated with both neutrophils and eosinophils in the airways, while bacterial colonization of the airways in stable neutrophilic corticosteroid-refractory severe asthma has been consistently reported. Specific pathogens may promote corticosteroid insensitivity as well as may enhance neutrophil survival in the airways (98). Many studies associated sputum neutrophilia with disease severity, accounting for 60% of patients with NA. In a subgroup of patients characterized by isolated sputum neutrophilia a poor response to ICS in terms of symptom scores, FEV1, and PC20 fall (99). On the other hand, neutrophils seem to protect against severe AHR in mild to moderate steroid-naive asthma, suggesting a different role for these cells in this setting of asthmatics compared to severe neutrophilic asthma.

#### Paucigranulocytic phenotype of asthma

The paucigranulocytic phenotype of asthma accounts for 38–52% of patients and has been associated with different clinical features: younger age, high BMI, better lung function, better control, less severe refractory asthma, and less often high doses of ICS compared to EA and mixed phenotype (69). Interestingly in paucigranulocytic patients, and in particular in steroid naïve, there was an increase in S-EOS counts compared to healthy subjects, pointing to low-grade inflammation in the so-called “non-eosinophilic phenotypes” of asthma, which may be corticosteroid sensitive (68). However, in some cohorts, there is a proportion of paucigranulocytic patients that remain uncontrolled, and that therefore may become refractory to treatment.

#### Sputum eosinophils biomarker of phenotype in severe asthma

The advent of asthma-biased and unbiased cluster analysis applied to asthma phenotyping has allowed the matching of the sputum inflammatory patterns with clinical, physiological, and biological characteristics of patients with severe asthma. The SARP cohort identified five clusters of which three gathered the majority of severe asthmatic patients (100); from the Leicester UK cohort, four severe asthma clusters emerged differing in symptom expression and sputum inflammatory cell characteristics (101); the U-BIOPRED cohort published by Lefaudeux et al. integrated transcriptomic signatures into the analysis identifying four reproducible and stable clusters of asthmatic patients (102). Taking into account the significant differences in the cohorts examined, an attempt in comparing the results coming from the above studies resulted in the identification of three main clusters of severe asthma: (1) early-onset allergic moderate-to-severe remodeled asthma, (2) late-onset nonallergic eosinophilic asthma, and (3) late-onset noneosinophilic nonallergic asthma (4). Elevated S-EOS is a common feature of both the early-onset, severe allergic, and late-onset severe nonallergic eosinophilic asthma, and the presence of S-EOS ≥2% is reported by the global strategy for asthma management and prevention (GINA) 2019 update as criteria to identify patients with severe asthma with refractory T2 inflammation despite high-dose ICS or daily OCS treatment. Anyway, S-EOS alone is not sufficient for the distinction between the two groups. In the severe allergic group, a higher count of sputum neutrophils is also detectable and is often associated with increases in total IgE, FENO, and B-EOS, and the presence of reversible severe reductions in pulmonary function, frequent exacerbations with seasonal variations with the need for OCS bursts. On the other hand, the late-onset nonatopic eosinophilic group has a higher degree of S-EOS, often associated with high B-EOS, severe nasal polyps (NP), aspirin sensitivity, frequent exacerbations requiring healthcare use, and may require OCS maintenance therapy for symptom control (103). The third common group of severe asthma patients from the cluster analysis is the late-onset, sputum neutrophil predominant. Herein the main features are the low atopic state, frequent symptoms, fixed airflow obstruction, OCS bursts, and HCU for exacerbations often associated with recurrent respiratory tract infections.

#### Sputum eosinophil biomarkers in refractory severe eosinophilic asthma

Refractory asthma may be defined as the requirement of maintenance of OCS to maintain control or prevent severe exacerbations. A specific inflammatory cell pattern is not associated with this clinical phenotype, yet while reducing OCS dose, an increase in S-EOS preceded the worsening of symptoms and FEV1, but clinical exacerbations were accompanied by sputum neutrophilia (104). According to an expanded data analysis from the SARP study, eosinophilic refractory asthma could be further split into two different clusters, the first characterized by late-onset severe asthma with NP and eosinophilia more prone to respond to corticosteroid treatment, even if rapidly deteriorated after discontinuation (corticosteroid dependent), and the second cluster with persistent inflammation in the blood and BAL, increased FENO levels, exacerbations despite high systemic corticosteroid use and side effects with signs of corticosteroid complete insensitivity (105). Even if mechanisms underlying steroid resistance are far from being elucidated, S-EOS reserved the best ROC curve among T2 biomarkers for the outcome of steroid-resistant T2-high patients, indicating that in a subgroup of severe asthmatic patients incompletely suppressed type-2 inflammation is a mechanism of severe asthma (106).

#### Sputum in real life and registries

Even if highly informative, sputum induction for assessment of cellular inflammation in asthma is seldom adopted in clinical practice, often limited to tertiary centers with high expertise. Real-life experience is limited, therefore lowering the power of this methodology in the complex evaluation of single patients. A retrospective analysis of asthmatics from a secondary care center confirmed the dependence of S-EOS on initiating or withdrawing ICS treatment and that only in the eosinophilic phenotype (sputum eosinophils ≥3%) did the effectiveness of ICS in improving asthma control, quality of life, FEV1, AHR, and exacerbation rate could be observed (107). The same authors reported that in two-thirds of 36 non-eosinophilic asthmatics, defined by S-EOS < 3% and B-EOS < 400/μL, withdrawing or reducing the dose of ICS was feasible, failing in those with an elevated B-EOS count (108). The BREATHE study, provided sputum data of 419 patients with asthma, resulting in mean S-EOS of 1.5%, and confirming the distribution of sputum inflammatory phenotype as reported in other studies [eosinophilic 28%, neutrophilic 18%, mixed inflammation 10%, and paucigranolocytic 44% (109)]. In the Wessex AsThma CoHort of difficult asthma, a multidimensional algorithm to determine the probability of an eosinophilic phenotype within a severe asthma population has been applied and resulted in 45% of the population being considered “most likely eosinophilic”. Interestingly no differences were observed in asthma control or the number of annual exacerbations, while older age, higher FENO, and later disease onset were found in the eosinophilic group. Unfortunately, 46% of patients classified as Grade 3 (eosinophilic) did not have evidence of S-EOS, while 25% of those identified as eosinophilic by sputum measures in WATCH also had neutrophilic airway disease. Finally, longitudinal repeated measures are needed for understanding the phenotype and confirming the prognostic and diagnostic value of sputum (110).

#### Sputum cells biomarker of biologic treatment

The recognition of the endotypes represented the biological basis for the use of monoclonal antibodies blocking the different immunological pathways. Application of sputum analysis in the clinical trials of biologics in asthma is yet very limited, being preferred its surrogate biomarkers. None of the 26 trials included in a meta-analysis of omalizumab treatment of severe asthma both in adults and children included the inflammatory sputum analysis (111). Djukanović and colleagues explored the effect of omalizumab in forty-five patients with mild to moderate persistent asthma with S-EOS of >2%, showing a significant decrease in S-EOS count from 6.6 to 1.7% in the omalizumab group compared to the placebo group. Moreover, they confirmed the anti-inflammatory effects of omalizumab treatment by assessing the reduction of tissue eosinophils, and high-affinity Fc receptor for IgE and T cell infiltrates (112). An observational study including 16 Japanese patients with severe asthma allergic to HDM showed changes in clinical scores for S-EOS (113) while real-life retrospective observations did not find S-EOS to be a predictor of response to therapy with omalizumab in patients with severe allergic asthma (114). Overall, the role of S-EOS as a biomarker in omalizumab-treated patients is poorly depicted. Sputum assessment has been included in some of the registrative trials for mepolizumab and reslizumab. Haldar and co-authors showed in refractory eosinophilic asthma, defined as an S-EOS ≥3% on at least one occasion in the previous 2 years despite high-dose corticosteroid treatment, that mepolizumab 750 mg i.v., monthly significantly lowered S-EOS count by a factor of 7.1. In addition during an exacerbation, a significantly lower mean S-EOS percentage in the mepolizumab group was observed compared to placebo (1.5 vs. 4.4%) (39). A subsequent meta-analysis about the efficacy of anti-IL5 therapy in patients with asthma confirmed the effects of mepolizumab on S-EOS% (115). The subsequent DREAM study enrolled patients with refractory eosinophilic asthma despite a high dose of ICS; in the subgroup of 94 patients who had sputum induction, mepolizumab, at three different dosages i.v., caused a significant decrease in S-EOS counts compared with placebo (41). On the contrary, the characterization of the clinical efficacy of a subcutaneous dose of mepolizumab was applied by selecting eosinophils count of >300 cells/μL as the eosinophilic phenotype of severe asthma (116) as well the MENSA TRIAL extended this approach by evaluating the efficacy of a 75-mg intravenous dose or a 100-mg subcutaneous dose of mepolizumab (42). The efficacy of mepolizumab in other trials was finalized to a different endpoint, such as QoL, OCS sparing effect, adverse events (MENSA; SIRIUS; COSMOS) and their extensions (COSMEX) did not include S-EOS count in the analysis. The recent MEX study, a multicenter, prospective, observational cohort study compared the characteristics of patients who had exacerbations on mepolizumab with those who did not, evidencing the differences among exacerbations with high S-EOS count (≥2%) from that with a low S-EOS count (< 2%) (117). As to real-world experiences, the treatment of 116 severe eosinophilic asthmatics with mepolizumab for at least 18 months resulted in FEV1 improvement in patients with a higher baseline S-EOS and a significant reduction in S-EOS counts by 60% after 6 months (118). Only another small study including 32 reported a reduction in S-EOS in patients with severe asthma treated with mepolizumab, of which 50% showed co-presence of bronchiectasis (46). Phase 3 Clinical trials evaluating the efficacy (SIROCCO, CALIMA), OCS sparing effect (ZONDA) and safety (BORA) of benralizumab for patients with severe uncontrolled asthma did not take into account S-EOS for inclusion criteria (119), nor did others Phase III studies with benralizumab exploring different outcomes (120). Just in a multicenter, double-blind, placebo-controlled phase I study, the effect of either single-dose intravenous or multiple subcutaneous benralizumab was evaluated on S-EOS count accounting for a high degree reduction (48), while a phase2b study reported the cut-off of 2% of s-EOS as inclusion criteria, but none of the outcomes included sputum cells count analysis (121). Evidence of real-life experiences of evaluating sputum inflammatory cells under benralizumab treatment is absent.

The Randomized Controlled Trial from Sally Wenzel enrolled patients with persistent, moderate-to-severe asthma and a B-EOS count of at least 300 cells/μl or an S-EOS level of at least 3%. Results of dupilumab on S-EOS levels were available for only 15 patients without giving any informative results (122). The severity measures for the efficacy of dupilumab evaluated in a meta-analysis of clinical trials did not include sputum cell count (123). A systematic review and meta-analysis evaluating all together with the real-world efficacy of treatment with benralizumab, dupilumab, mepolizumab, and reslizumab for severe asthma did not evidence a role of sputum induction (124).

#### Variability over time of sputum

Sputum cellularity has been mainly used as a single-point tool to identify a patient with severe asthma as having an eosinophilic phenotype in cross-sectional measurements. The utility of repeating sputum cell count in the contest of trials let to optimize the response to ICS or the efficacy endpoint in trials for biologics. However, the longitudinal stability of phenotypic sputum clusters is debated and may influence the use of this biomarker in clinical practice. The Pan-European BIOAIR study of severe asthma reported sputum phenotypes at baseline and 1 year of follow-up according to the four subgroups identified by Hastie et al. (percentage of eosinophils < 2 or ≥2%) and neutrophils < 40 or ≥40%). After 1 year of follow-up of 52 patients, 42.3 and 48.6 of the whole asthma population and severe asthmatics changed the allocation to particular clusters, respectively. Interestingly the phenotype change was not influenced either by the dose of inhaled corticosteroids or orally used either in phenotypes or by the number of exacerbations reported during this time (72). Repeated measurements at different thresholds of S-EOS were retrospectively analyzed in a group of severe asthmatics: 47.4% of participants (*n* = 87) consistently exceeded S-EOS > 3%, while other 47.4% (*N* = 82) were crossing the threshold along the time of observation (34). Generally, reports assessing longitudinal stability of sputum airway inflammation show a very wide range of longitudinal persistence of elevated eosinophils in both moderate and severe asthma (7 to 76%). Moreover, the time intervals of sputum collection vary greatly, ranging from 1 month to 5 years. High variation in S-EOS in subjects with severe asthma was associated with a twice loss of lung function over 8 years compared to patients with persistently non-eosinophilia or persistently eosinophilic asthma (125). The examination of both S-EOS and neutrophils for longitudinal variation associated with pulmonary function and healthcare use within the SARP III cohort resulted in the lowest prebronchodilator FEV1% being associated with a combination of predominantly ≥2% eosinophil and ≥50% neutrophil groups (mixed cells).

#### Clinical applicability of sputum cellularity

In the context of allergic asthma, S-EOS count did not find a routine application to evaluate exposure to allergens nor to predict AHR and the very rare use of allergen-specific challenges limited to research aims reduces sputum applicability to very limited perspectives such as response to immunotherapy. Cross-sectional studies of wide asthmatic populations confirmed sputum cellularity as a parameter to guide corticosteroid treatment and the prediction of exacerbations. This kind of use may become decisive in discriminating the type-2 inflammation component during concomitant infected bronchiectasis or viral superinfections, giving additional information from FENO and acute phase markers. On the other hand, the ability of sputum cells to capture disease control or severity varies extremely according to the asthma population to which it is applied. To be taken into account the lack of consistency in sputum cellular cut-offs and that at the moment the real-life assessment of sputum cellularity is limited to tertiary centers. It is becoming a great impact the application of sputum analysis in phenotyping asthma patients between eosinophilic and non-eosinophilic; such differentiation can help in predicting the natural history of the disease, the lung function decline, and the response to corticosteroid treatment. Cluster analysis led to the identification of main clusters of severe asthma in any of which the sputum cellularity is peculiar, even if not exclusive. The progressive capacity of identifying the phenotypes by matching sputum analysis with clinical traits allows the understanding of how the patients will respond to corticosteroids, therefore predicting cumulative side effects, and biological treatments, while the “a priori” choice of biologic remains a goal still to be cached. Sputum analysis, however, is not able to differentiate the overlap between severe asthma phenotype and the concurrent use of other biomarkers in mandatory.

### Exhaled nitric oxide (FENO)

#### FENO methodology and normal range

The value of measuring FENO concentration in asthma emerged in the last 20 years thanks to its function as a marker of airway inflammation and the non-invasive, reproducible, and sensitive technique of its measurement. The utility of FENO in the management of asthma has been validated in different settings including diagnosis, treatment response and adherence, and asthma phenotyping (126). Guidelines for FENO measurement by The American Thoracic Society (ATS) and the European Respiratory Society (ERS) have standardized the procedure recommending an exhaled flow of 50 ml/s (FEno50) (127). Healthy individuals have FEno50 values between 10 and 20 ppb, yet Kharitonov et al. published data indicating that the upper limit, 2SD above the mean for healthy individuals, is 33 ppb and these results have been confirmed by a very large survey by the National Health and Nutrition Examination Survey (NHANES) reporting the fifth to 95th percentile values of FENO being 3.5–39 ppb for subjects 12–80 years of age (128). As it is assumed that FENO values can be influenced by several non-disease-related factors, such as diet, medicine intake, current smoking, and atopy, the application of multiple regression modeling reported normal values of FENO in never-smoking adults, irrespective of the presence of atopy, ranging from 24 to 53 ppb. Another study found that FENO levels were similar in never-smokers and former smokers (128) (Table 6). All these data show that the distribution of FENO in an unselected population is skewed to the right, therefore it is unlikely that reference values derived from a “normal” population will be as helpful as cut points in patients with airway disease or respiratory symptoms (131).

**Table 6:** Reference values of FENO in healthy subjects.

	**Healthy adults**		**Healthy children**	
	**Geometric mean (ppb) -**	**5–95th percentile**	**Geometric mean (ppb) (95% CI)**	**5–95th percentile**
Kharitonov et al. (132)	17.8	[2–31.4]	15.6	[0–34]
See and Christiani (128)	13.3	[3.5–39]	9.0	[3.5–36.5]
Olin et al. (129)	16–6	[5.87–47,14]		
	**Never/ex smokers**		**smokers**	
Torén et al. (130)	15.7	[7.8–35.7]	10.4	[4.4–29.4]
	**Atopic subjects**		**Non atopic subjects**	
Olin et al. (129)	18.8	[6.03–58.74]	16.0	[5.91–58.76]

#### FENO cut-offs for eosinophilic inflammation in asthma

A good correlation between FENO values and objective evaluation of airway inflammation by major basic protein (MBP) of bronchial biopsies and by eosinophils count in bronchoalveolar lavage of children with mild-to-moderate asthma has been reported (133) and a correlation between S-EOS and FENO, even if not linear, had been shown (134). Repeated allergen exposure caused a gradual increase in FENO with a peak after 7–10 days following the 4 days increases in the percentage of S-EOS (135). The cut point for FENO that best fit with an S-EOS count of ≥3% was 21 ppb in corticosteroid-naïve patients, while in a trial designed to titrate by FENO corticosteroid dose in a primary care setting of asthma, A FENO of < 26 ppb was associated with a differential S-EOS count of < 3% for 85% of all visits (136). The large study by Berry reported that FENO50 of 36 ppb had a sensitivity and specificity for S-EOS of more than 3% of 78 and 72%, respectively. The 2011 ATS guidelines recommend a mean FENO of 20 ppb for subjects aged 6–11 y and 25 ppb for subjects aged 12–80 y as a marker of less likely eosinophilic airway inflammation in asthma; a mean FENO of 35 ppb for subjects aged 6–11 y and 50 ppb for subjects aged 12–80 y as highly connected with airway eosinophilic inflammation; and FENO values between 25 and 50 ppb (20–35 ppb in children) to be interpreted cautiously according to the clinical context (131).

#### Correlation between FENO, airway hyperresponsiveness, and diagnosis of asthma

Pieces of evidence regarding the correlation between FENO levels and AHR are conflicting, greatly varying according to the cohort of patients studied. In a population sample of 306 young adults who also underwent bronchial challenge with histamine, FENO correlated significantly with AHR and was significantly greater in asthmatic subjects (mean, 22.2 ppb) than in normal subjects (7.8 ppb). When applying FENO as a tool to test adult patients with suspected bronchial asthma, it allowed for discrimination, among patients, and without AHR, in steroid-naive patients, while in asthmatic subjects treated with ICS there was no relationship (137). The specificity and sensibility of FENO in the diagnosis of asthma vary between studies depending on the pre-test probability; often asthmatic patients suffer from chronic rhinitis which is an independent risk factor for rising FENO levels; accordingly, Heffler and coworkers reported a cut-off point of FENO >36 ppb associated with the highest combination of specificity (60.0%) and sensitivity (77.8%) for patients with asthma symptoms and concomitant persistent rhinitis (138), rising to >42 ppb when patients with asthma symptoms and chronic rhinosinusitis were included. On the other hand, FENO < 25 ppb has a very high negative predictive value, being, therefore, able to rule out the diagnosis of asthma. The ATS guidelines suggest, with a weak recommendation, that FENO may be used to support the diagnosis of asthma in situations in which objective evidence is needed. According to the British National Institute for Health Care Excellence (NICE) guidelines, a relevant population for the assessment of FENO in the diagnosis of asthma in patients aged ≥5 years presenting to primary care; people who are difficult to diagnose due to confusing factors such as obesity, anxiety; patients who may experience different outcomes such as smokers, the elderly and pregnant women. In addition, FENO measurement could also be used in differentiating Cough-Variant Asthma from other causes of chronic cough (139).

#### FENO biomarkers of exacerbations, steroid responsiveness, and asthma control

The increase in FENO levels has been shown to predict asthma exacerbations. After ICS withdrawal in patients with moderate asthma the development of symptoms and S-EOS correlated with changes in FENO. An increase in FENO > 60% was able to predict 1 week before, loss of asthma control with positive predictive values between 80 and 90% (140). In a prospective study of 44 nonsmoking asthmatics clinically stable for 6 weeks and receiving 250 mcg of fluticasone/50 mcg of salmeterol or equivalent for 3 years, a baseline FENO ≥28 ppb was able to predict the first exacerbations with a probability of 76% ad a relative risk for exacerbation of 3.4 (141). Therefore, different FENO cut-offs are applicable for the prediction of exacerbation depending on whether the patients are steroid naïve or treated. The opposite aspect of the same phenomenon is the likelihood of FENO to predict corticosteroid responsiveness. ICS reduces FENO in asthma as compared to placebo in a dose-dependent manner (142). The consequence is the capacity of FENO to predict poor asthma control. Following steroid withdrawal, both single measurements and changes of FENO (15 ppb change or an increase of >60% over baseline) had 80 to 90% positive predictive values for predicting a loss of control. Even if FENO had similar results compared to similar S-EOS and saline PD (15) measurements, as well as bronchodilator use and symptoms, the advantage consisted of the easiness of performance and the rapidity of effect (140). Yet in clinical practice, in which ICS is not abruptly suspended, the correlation between FENO levels and asthma control resulted in contradictions as reported by a meta-analysis (143) being better in patients not on regular treatment for asthma and worst in patients regularly treated with ICS. The influence of comorbidities such as sino-nasal disease has to be taken into account, due to the capacity of influencing both asthma control and levels of FENO (123). Other factors influencing the FENO values over asthma control are smoking and atopy. Smoking reduces FENO values by a 20% quote, but sequential changes in FENO have a relationship with asthma control (144). The application of FENO to reflect asthma control in the regular clinical setting of 341 unselected asthma patients resulted in a FENO decrease of < 40% as a sign of asthma control optimization, being significant only in patients in low-dose and not in high-dose ICS. On the other hand, an increase in >30% of FENO correlated to LOC (145). The Long-Acting Beta-Agonist Step-Down Study (LASST) was designed to assess whether Type 2 markers predicted the time to loss of asthma control among well-controlled asthmatics during step down, a continuation of stable dose, or interruption of therapy. All the three groups had significant rates of treatment failure of about 30%, but serially measured FENO stratified on three levels (< 25 ppb; 25–50 ppb; >50 ppb) did not have a significantly increased likelihood of subsequent treatment failure (146).

Another consequence is the importance of FENO to identify steroid responsiveness that is clinically relevant enabling the clinician to bypass an empiric “trial of steroids” or unnecessary long-term corticosteroid treatment. In ICS-naïve patients, FENO >35 ppb predicted asthma control improvement in response to ICS, but others reported the optimum cut point was 47 ppb. Moreover, the likelihood of relapse following the 4 weeks of withdrawal from ICS therapy is greatest in patients reporting FENO to increase above 49 ppb (146). The ATS recommendations state that for FENO > 50 ppb (>35 ppb in children) responsiveness to corticosteroids is likely. Conversely reaching low FENO (22 ppb) predicts the likelihood of successful reduction or withdrawal of ICS (positive predictive value, 92%). The assessment of low FENO (25 ppb,20 ppb in children) is in symptomatic patients, on the other hand, a suggestion for responsiveness to corticosteroids is less likely. Noneosinophilic asthma (probably steroid unresponsive) or additional or alternative diagnoses have to be ruled out.

#### Monitoring asthma treatment and adherence with FENO

FENO has been proven to decrease rapidly after treatment, more quickly than other markers such as lung function parameters, symptoms, or airway hyperreactivity, and to increase rapidly before worsening asthma control and exacerbations. A study involving eighty-five children with atopic asthma demonstrated that a treatment decision combining both FENO and symptoms improved more hyperresponsiveness than in the symptom group. The trial conducted by Smith et al with treatment adjusted based on either FENO measurements or an algorithm based on conventional guidelines found that FENO tailored group had a significantly reduced final mean daily doses of fluticasone as well as a reduced number of exacerbations (147). A review including 5 trials for adults reported no difference between the two intervention groups in major or severe exacerbations and OCS intake, while it was significant in a composite outcome of major/severe, moderate, and minor exacerbation rates/treatment failures. Moreover, ICS use remained the same or felt in FENO-managed groups. No effect on health-related quality of life and other medication use could be observed (148). In a more recent review, Petsky et al., including seven adult studies, reported that the number of people having one or more asthma exacerbations was significantly lower in the FENO group compared to the control group (OR.6), but no difference was found for exacerbations requiring hospitalization or rescue OCS or for any the secondary outcomes including FEV1, symptoms scores, ICS doses (149). Nonadherence to ICS is a major cause of poor control, and the different ways to unveil this behavior have been hypnotized. FENO is a possible tool to identify nonadherence and a “FENO suppression test”, has been proposed by administering inhaled budesonide 1,600 mg for 7 consecutive days to asthmatic patients with persistently elevated FENO despite treatment and measuring FENO for 8 days and weekly for 4 weeks. Suppression of FENO could predict 92% in nonadherent patients. The applicability in routine clinical care, coupled with remote monitoring technology, was confirmed (150). We think that increased employment of FENO in general clinical practice, not limited to highly specialized asthma for asthma care, is valuable to obtaining the best adherence and preventing uncontrolled disease.

#### FENO cluster analysis of asthma phenotypes

A FENO level >20 ppb is suggested by the GINA criteria for type 2 inflammation. In The SARP study, FENO levels are similar in all six clusters (range of median 24.8–32.8 ppb), including the group with a higher fraction of patients with severe asthma (100). Dividing the SARP population into mild, moderate, or severe asthma no substantial differences in FENO were observed. When a machine learning approach was applied to the SARP population, cluster 6 of severe asthmatics had higher FENO values compared with all other clusters, being characterized by early-onset, most symptoms, the lowest lung function, frequent and high-intensity HCU, and sinusitis (105). High FENO values (>50 ppb) in asthma were reported correlating to the higher number of exacerbations, the more when combined with a high blood eosinophils count (151). In the Leicester refractory asthma, FENO values among the identified four severe asthma clusters resulted higher in cluster 1 (Early Onset Atopic) and cluster 4 (Inflammation Predominant) at 51.2 and 52.1 ppb respectively, which had also the higher eosinophilic inflammation (101). U-BIOPRED, stratified by clinicophysiologic parameters and omics sputum analysis, patients with moderate-to-severe asthma, presented 4 clusters not differentiated for FENO values (mean range 24–33.8 ppb). However, filtering on the differentially expressed genes between eosinophil and noneosinophil-associated, yielded three groups (TACs) the first of them (TAC1) being characterized by the highest FENO (mean 29,5 ppb) associated with high serum periostin, eosinophilia, high OCS dependency, acute exacerbation, NP and severe airflow obstruction (152). An alternative transcriptome gene cluster analysis from bronchial biopsies and epithelial brushings of 107 U-BIOPRED subjects identified, two subgroups with eosinophilic inflammation and steroid insensitivity, one with the highest FENO levels (FENO 56.5 ppb), frequent acute exacerbation rate, and OCS use, and intermediate to high S-EOS, and the second with the highest levels of S-EOS, high BMI, intermediate to high FENO (35.5 ppb) (153). Another Unsupervised clustering analysis based on sputum gene transcriptomic expression identified three clusters of endotypes (TEA), among which TEA1 and TEA 2 has significantly FENO higher level of TEA 3 (53 and 52 vs. 38ppb), the latter being characterized by milder asthma, the lowest daily ICS dose, preserved lung function and minimal bronchodilator reversibility (153).

#### FENO in severe asthma: Registry and real-life studies

The measure of FENO in a clinical routinary setting only partially confirmed the identification of different subgroups as suggested by cluster analysis. The real-life experience from the Italian severe asthma registry (SANI) reported a mean value FENO of 48.0 ppb, with a distribution of patients according to FENO clustered >25 ppb (40.9%) or more than >40 ppb (40.9%). The higher FENO values were in patients with CRSwNP (mean FENO 54.5 ppb). Interestingly reclassifying the patients according to a different definition of severe asthma, the subgroup that met the ATS/GINA criteria for severe asthma had a higher FENO level than the others, suggesting the capacity of this biomarker to select severe asthmatics among patients sharing some degree of severity (9). The data from the ISAR showed that, overall, 43.1% of patients with severe asthma had FENO concentrations < 25 ppb, while FENO >50 ppb was reported in about 30% of cases (154). Stratifying patients by these FENO cutoffs to a real-world cross-sectional series showed that specific asthma phenotypes with distinct clinical features can be identified (155).

#### FENO and biologics: Clinical trials and real-life studies

**Figure 1:**
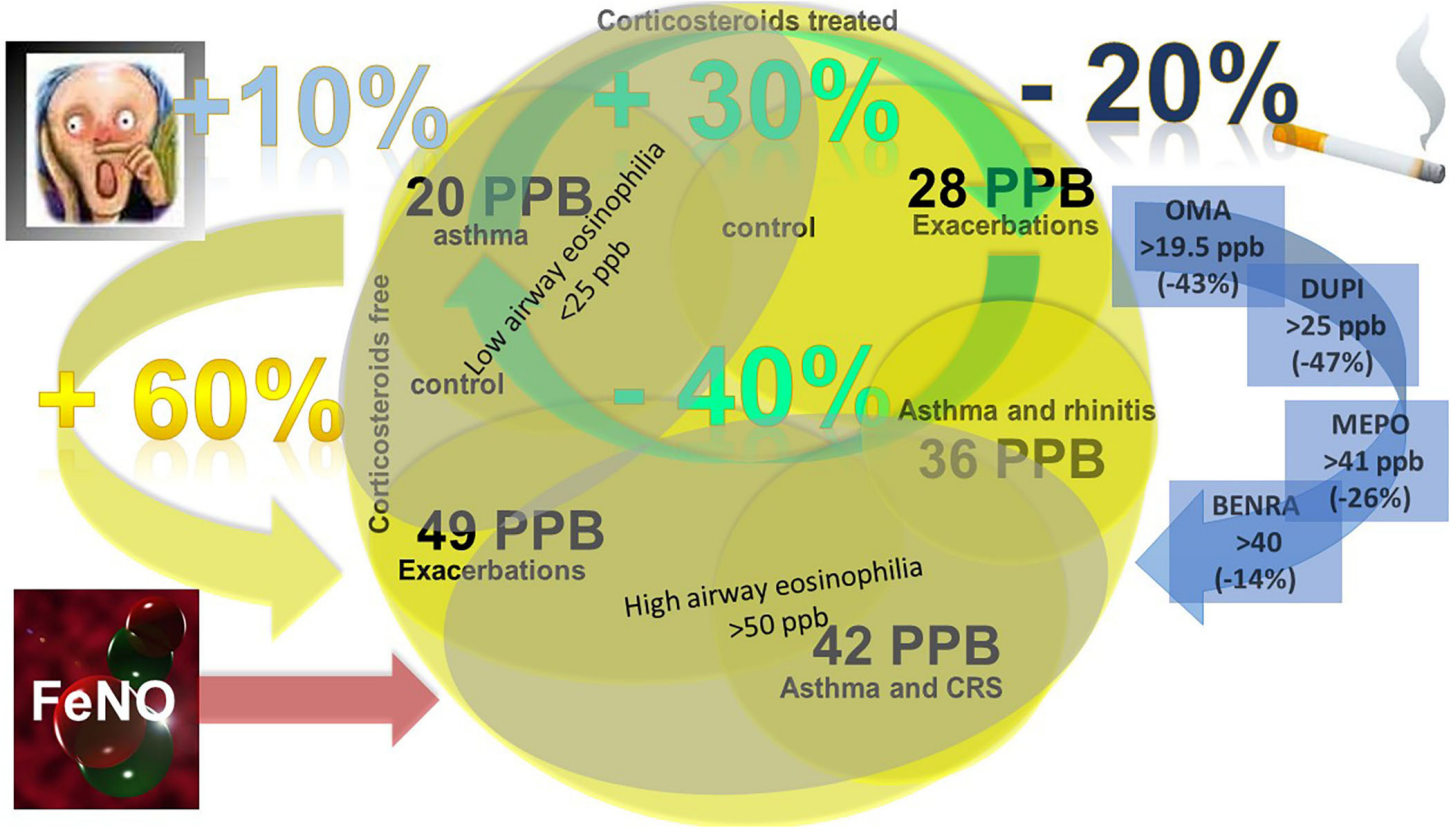
FENO cut-off levels in asthma clinical practice. FENO >20 ppb for asthma diagnosis (88% sensitivity and 79% specificity); FENO > 36 and >42 ppb for asthma diagnosis and concomitant rhinitis and CRS, respectively; FENO < 25 ppb less likely for airway eosinophilia; FENO >50 ppb more likely for airway eosinophilia and ICS responsiveness; FENO >28 ppb and >49 ppb predicts exacerbations in steroid-treated and naïve patients, respectively; FENO increase +60% predicts loss of control after steroid withdrawal; FENO increase +30% predicts loss of control in steroid-treated patients; FENO decrease −40% predicts good control in steroid-treated patients; when assessing asthma control/exacerbation smoking habit reduce the quote of FENO of −20%, rhinitis increase of +10%; the FENO values predictors of best response to biologics and the FENO reduction expected (%) are reported (OMA, omalizumab; DUPI, dupilumab; MEPO, mepolizumab; BENRA, benralizumab).

The use of FENO as a biomarker for severe allergic asthma treated with omalizumab is limited in clinical trials. Hanania and colleagues reported a mean FENO of 29.5 ppb and observed in a subgroup of 394 patients during 48 weeks, a greater reduction in FENO from baseline in the omalizumab group with a reduced capacity of −4.24 ppb, compared with the placebo group (156). The EXTRA Study explored the effects of Omalizumab in Allergic Asthma by the analysis of biomarkers and showed in the high FENO subgroup (>19.5 ppb) a greater reduction in exacerbations vs. the placebo group compared to the low FENO (< 19.5 ppb), 53 vs. 16% respectively. The XPORT randomized, double-blind, placebo-controlled trial, evaluating the benefit and persistence of response in patients continuing or withdrawing omalizumab after long-term treatment, revealed the capacity of an increase in FENO values from baseline to week 12 after omalizumab discontinuation to predict exacerbations (61). A systematic review of clinical trials and real-world experiences of omalizumab in moderate-to-severe uncontrolled asthma in children, reported only one study exploring the effect of FENO, reducing in the omalizumab group (from 41.9 to 18 ppb) (157). Comparing clinical outcomes and biomarkers in patients with moderate-to-severe allergic asthma who have been prescribed omalizumab (ASTERIX) after 12 months of treatment, FENO was reduced from 47.3 to 37.1 ppb; the total annual OCS dose and the number of exacerbations reduced mainly in a high FENO baseline group (>19.5 ppb) (158). The Xolair Italian study group found no differences in FENO outcome in 4 groups divided according to the duration of treatment from 12 to 60 months (159). Among the key secondary endpoints of the QUEST Study, a phase 3 trial of the efficacy and safety of dupilumab in moderate to severe uncontrolled asthma, severe exacerbation rates reduced in patients with baseline FENO levels ≥25 ppb and even more for values ≥50 ppb and the improvement in lung function was significant for baseline FENO levels ≥ 25 ppb. The pharmacodynamic effect of Dupilumab on FENO showed a rapid reduction after 12 weeks, sustained at 52 weeks at a 47% of decrease (160). In the LIBERTY ASTHMA QUEST, the efficacy of dupilumab in reducing the exacerbations was significantly higher in those with baseline FENO >25 ppb, independently from the atopic status. Moreover, after 2 weeks of treatment, FENO started to decrease, with this effect sustained throughout the 52-week treatment period, falling to a mean of 16 ppb (62). Patients enrolled in the VENTURE trial had a mean FENO level of 37 ppb despite the use of oral glucocorticoids in the last 6 months (5–35 mg/day of prednisone); 79% of patients in the active arm received 300 mg of Dupilumab every 2 weeks and presenting with baseline FENO ≥25 ppb, obtained a reduction of OCS to 5 mg (160). The open-label study derived from both QUEST and LIBERTY, named TRAVERSE, and extended to 96 weeks, did not report data about FENO. A 4-step approach was carried out to calculate the dupilumab-eligible population, resulting in that raised levels of both FENO and B-EOS that could occur in 52% of severe asthmatics, while FENO > 25 ppb alone in a smaller portion of patients (11%) (161). The few real-life experiences reported early improvement in FENO after only 3 months of dupilumab biologic therapy. It was addressed that the introduction of FENO evaluation in the selection criteria for dupilumab, helps the identification of eligible patients among type-2 severe asthma patients and allows a complete outpatient assessment (162). Another experience reported in 20 patients with severe asthma and NP was a great reduction in FENO after 4 weeks of treatment with dupilumab (from 27.5 to 2.5 ppb) (163). FENO has not been extensively explored in the registrative trials for mepolizumab. Haldar reported a mean FENO value of 44.4 and 35.5 ppb in the treated and placebo groups respectively, but there were no significant between-group differences in the change in FENO ad the end of treatment (39). As a proof of concept, a study elicited airway inflammation in mild allergic asthma by segmental allergen challenge before and 1 month after a single intravenous 750-mg dose of mepolizumab, reporting non no differences in the pre and post-challenge FENO values after mepolizumab treatment (164). The DREAM study reported basal FENO values of patients at about 30 ppb in all three arms and the placebo group. The effect of mepolizumab, in terms of the ratio of geometric mean FENO either to baseline or to placebo, was not significant (41). Neither MUSCA, MENSA, nor SIRUS trials evaluated FENO levels, as well as all the *post hoc* analyses derived from these studies. The majority of real-life studies confirmed a moderate quality for a reduction in FENO after treatment with mepolizumab (165) and when present, the reduction remained significantly above the normality (166) and generally reduced by a small fraction (26%). However, in a subgroup of patients non-responding to omalizumab, a switch to mepolizumab caused 50% in FENO levels (167). Patients who have an exceptionally good response to mepolizumab, termed “super-responders”, were characterized by higher FeNO (41 vs. 23 ppb) compared to patients with milder responses (168). FENO (≤ 20 or ≥50 ppb) was the most useful discriminator of inflammatory phenotype at exacerbation under mepolizumab treatment in the MEX study (117). The phase 2 trial, comparing different dosages of benralizumab in severe eosinophilic asthma, did not show any difference in FENO values after 52 weeks of treatment, while Phase 3 trials (SIROCCO, CALIMA, ZONDA, and BORA) did not take FENO into account (121). The real-life experience documented a significant reduction in FENO levels after 24 months of treatment, and this effect was much more evident in patients with CRS without NP (−45%) compared to those with NP (−10)(169). A meta-analysis reported overall a shred of low-quality evidence, indicating no significant change in FENO after treatment with benralizumab (mean reduction −14.18 ppb) (124). An experience in 21 patients reported the best cutoff value of 40 ppb for predicting response to benralizumab after 24 weeks (170). Figure 1 summarizes FENO cut-off levels in asthma clinical practice.

#### Stability over time of FENO

Few studies assessed the individual distribution and variability at different thresholds of FENO. Kharitonov reported very highly the repeatability and reproducibility of FENO measurement at different visits and time points (132), but the choice of the cut for high and low levels is crucial in evaluating the variability over time. One study reported that FENO levels persisted at values >19.5 or >25 ppb in more than half of the participants, but about 30% of participants crossed this threshold during different determinations (31). It is assumed that the within-subject coefficient of variation for FENO is 20% in patients with asthma. In conclusion, a personal best FENO50 should be estimated and used as the basis for evaluating treatment with anti-inflammatory drugs (171).

## Overlap between serum and local biomarkers

The clinical use of every single biomarker in asthma has to be carefully weighted according to the different relevant goals it demonstrated to satisfy in both trials and real-life experiences. However, a single biomarker is not able by itself to answer accurately to many clinical questions, including the ability to phenotype asthma. Therefore, the combined use of biomarkers is highly suggested to increase the specificity of the outcomes desired. Cluster analysis integrating data collection and multidimensional approaches to phenotype asthma, even if adding much knowledge to the endotypes underlying the heterogeneity of asthma, has not yielded a definite answer about the discovery of appropriate biomarkers for each phenotype. In clinical practice, several biomarkers exist for T2 inflammation above discussed but they do not discriminate well between endotypes or precisely predict a patient's response to a biologic (4). In the contest of moderate-to-severe asthma, *post-hoc* analyses have been applied using accepted definitions for allergic asthma (skin prick–positive and/or positive serum–specific IgE >0.35 kU/L), eosinophilic asthma (B-EOS high count ≥300 cells/μL) and type-2 asthma (FENO ≥35 ppb). A high degree of overlap was shown within each asthma population, therefore within the allergic asthma population 39.5 and 29.5% had eosinophilic asthma and Type-2 asthma, respectively; conversely, within the eosinophilic asthma population, 75.8 and 41.3% had, respectively, allergic asthma and type 2 asthma. Finally, the T2 asthma population accounted for an overlap of 81.1% of allergic asthma and 59.2% of eosinophilic asthma. The overlaps among subtypes increased at low cut-off values (eosinophils low cut-off ≥150 cells/μL and FENO low cut-off of ≥25 ppb) (172). Another study showed that 70% of patients with severe asthma had at least one type-2 biomarker elevated of which only 31% with a single biomarker, 39% with two or more elevated markers, and 15% had all three Type-2 biomarkers elevated (B-EOS count ≥0.3 x10^9^/L; elevated FENO ≥25 ppb; elevated IgE ≥150 × 10^3^). Interestingly concomitant elevation of 2 or more biomarkers was associated with a higher frequency of NP. This study took also into account sputum cellularity, with a prevalence of 43% of the patients with airway eosinophilia (eos ≥ 3%), but only 53% had concomitant airway and systemic eosinophilia. Both B-EOS and FENO could predict, by multiple logistic regression, S-EOS (AUC, 0.81 and 0.67; respectively). An attempt to set up a model for integrating the use of multiple biomarkers and their longitudinal variability has brought to identify four different clusters of patients. A Cluster 1, with a highly variable phenotype and high levels of T2 inflammation, had a low average level of B-EOS, therefore not representing the optimal initial treatment biomarker. A second cluster, with very low variability and high T2 inflammatory levels, could have beneficial effects from all the biologics targeting eosinophilic phenotypes. Cluster 3 gathers the non/low-T2 inflammatory phenotype, reporting the lowest B-EOS, FeNO, S-EOS, and IgE values. A fourth cluster, showing fluctuating Type-2 biomarkers around the threshold, will need multiple sensitive biomarkers assessment (34). The expression of biomarkers in severe asthmatics has been analyzed within the ISAR; although confirming a substantial overlap among inflammatory biomarkers, five distinct clusters exhibiting unique clinical characteristics were identified (5).

## Conclusions

We reviewed many of the findings concerning the use in the clinical practice of inflammatory biomarkers in asthma. Far from the scope of a systematic review, critical considerations about pathophysiological and methodological aspects have been matched with a step-by-step analysis of the unique contests in which a biomarker can be applied in clinical practice. Without underestimating the great thrust these biomarkers have given to asthma research and practical management, we have underlined the limits when using a biomarker for a clinical decision. Among the most questioning point concerning blood eosinophilia, the cut-offs we currently use for defining an eosinophilic inflammation (>300 or >150 × 10^6^/L) are considered, out of this contest, normal values of eosinophilia. Furthermore, the role of B-EOS as a surrogate biomarker of airway eosinophilia is contested by its low sensitivity and specificity. One of the consequences is the poor correlation between B-EOS and S-EOS at baseline in patients who entered a clinical trial of mepolizumab for severe eosinophilic asthma can have a heavy relapse in the clinical setting (173). Accordingly, a metanalysis assessing the diagnostic accuracy of FENO, B-EOS, and total IgE concluded moderate sensitivity and specificity leading to a substantial number of false-positive and false-negative (35). The cross-sectional analysis of B-EOS counts, FENO levels, and total IgE levels did not accurately predict S-EOS percentages, despite the significant association with S-EOS and the combinations of these variables did not improve the low predicting capacity (32). In a different clinical contest, however, a green traffic light comes on when combining FENO and B-EOS. This is the case of predicting the capacity of identifying patients at risk of exacerbations or poorly controlled patients (151). The management of severe asthma remains a daily challenge as there is not currently a universal shared algorithm for biomarkers application. An integrated approach including clinical and molecular phenotyping about responses to biologic therapy has been proposed, showing that the combination of high FENO or B-EOS, Age at onset, and clinical traits, such as nasal polyps, can make the decision more reliable (174). Among the ideal characteristic for a biomarker in FENO, IgE and B-EOS share the easy measurability, reproducibility in a clinical setting, and, to a different degree, a relationship to endotype mechanisms and targeting biologics. Sputum on the other hand, even if highly informative about the ability to provide information about prognosis and clinical outcome, is not routinely assessed except for tertiary centers of expertise, needing need specialized equipment and trained staff (22). However, its full potential in clinical practice is not yet discovered, considering the high prevalence of paucigranulocytic and mixed phenotypes that still deserve a comprehension of the immunologic and pathophysiologic mechanisms (175). Other candidate local and systemic biomarkers have been proposed by aiming to fill the gaps left by those described in this review. However, the advantages of these molecules, such as periostin, volatile organic compounds, sputum cytokines, and neutrophils seem at the moment very limited or still to be discovered (175) (Table 7). The road to the best clinical practice in asthma management will be the natural consequence of the correct application of the current biomarkers in the right clinical context (176, 177).

**Table 7:** Summary of asthma current biomarkers' characteristics.

**Biomarkers in clinical use**	**Collection methods and advantages**	**Clinical practice application**
Sputum eosinophils	Non invasive; Need of specialized equipment and trained staff; Not all samples can be adequate for processing	Provide a characterization of inflammatory status of the airways; Predicts responses to corticosteroids
Blood eosinophils	Minimal invasive; Easy to perform; Painful for some patients	One of criteria to define T2 high phenotype; Biomarker for the eligibility to anti-IL-5/anti-IL-5R treatment; Predict response to ICS and biologics (anti-IL-5/anti-IL-5R)
FeNO	Non invasive; User-friendly; Easy to collect	One of criteria to define T2 high phenotype; Describe the inflammatory status of the airways; Biomarker for the eligibility to anti IL-4R; Predict response to ICS and biologics
Serum IgE	Minimal invasive; Easy to perform, Painful for some patients	Biomarker for the eligibility to anti-IgE; Associated with specific sensitization to seasonal and perennial allergens; Associated with inflammatory, immunologic or hematologic disorders
**Other biomarkers**		
Sputum neutrophils	Non invasive; Need of specialized equipment and trained staff; Not all samples can be adequate for processing	Provide a characterization of inflammatory status of the airways
Serum Periostin	Minimal invasive; Easy to perform; Painful for some patients	Predicts a greater airway obstruction and lung function decline, Predicts therapeutic responses to ICS
Blood neutrophils	Minimal invasive; Easy to perform; Painful for some patients	Associated with symptom control and asthma exacerbation
Sputum Cytokine	Non invasive; Need of specialized equipment and trained staff	Characterize the inflammatory phenotypes
VOCs	Non invasive	Identify inflammatory phenotypes

FeNO, fractional exhaled nitric oxide; ICS, inhaled corticosteroids; VOCs, volatile organic compounds.

## Summary

**Figure 2:**
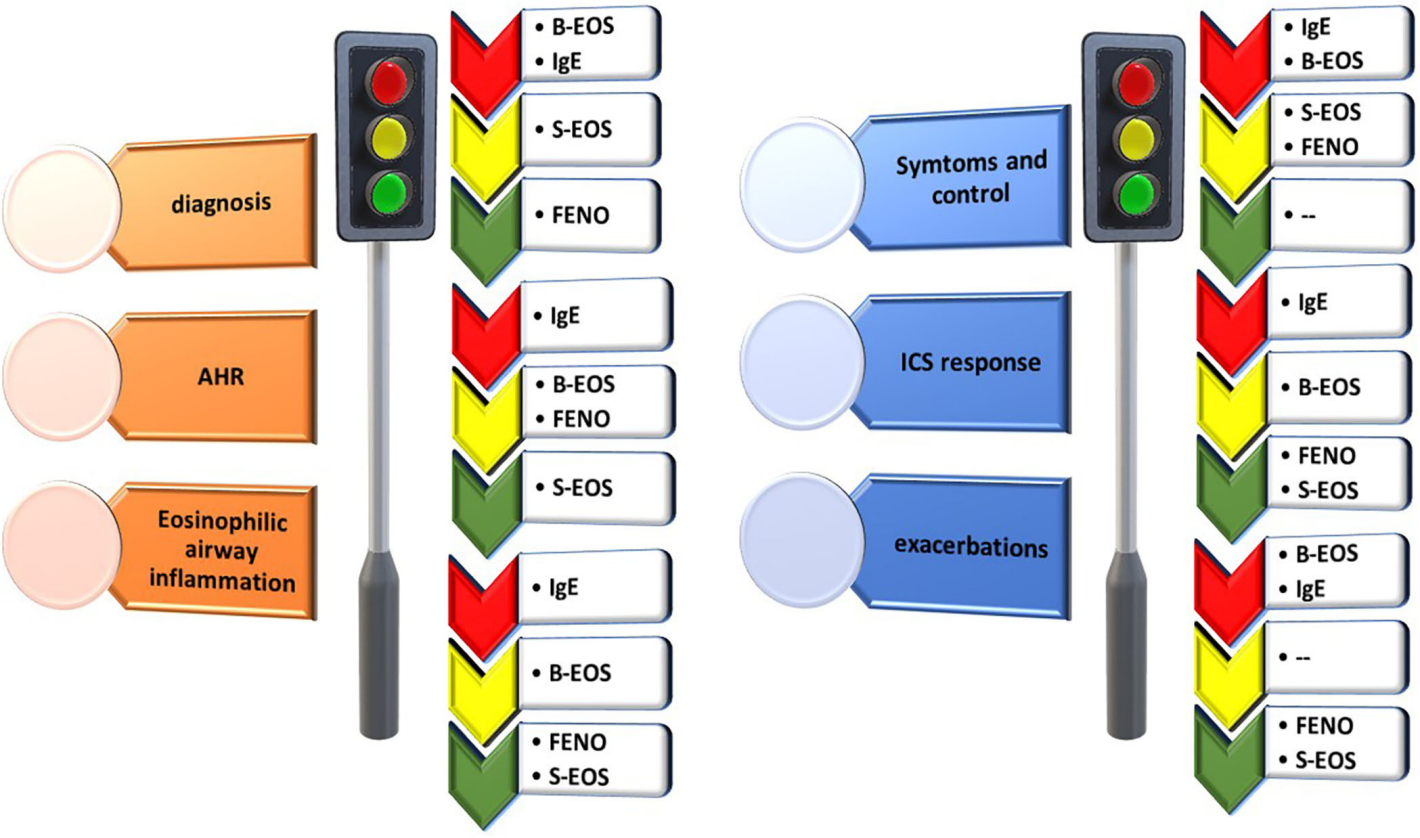
Strengths and weaknesses of asthma biomarkers in clinical practice. Red light: Poor evidence; yellow light: Debated/to be improved; green light: Good evidence.

Blood eosinophils, S-EOS, FENO, and total IgE are the main biomarkers routinely used in daily clinical practice for asthma diagnosis, phenotyping, and management. Each biomarker can retain strong, debated, or weak evidence in the different fields of applicability. FENO is helpful in asthma diagnosis and relates to eosinophilic airway inflammation. S-EOS has some evidence of association with AHR, depending on the asthma population and ongoing treatment, and is, as well, a good predictor of asthma exacerbations and response to ICS treatment. B-EOS can be used as surrogate markers of eosinophilic asthma inflammation, and even if it is the most accessible, gives not satisfying information about asthma control or treatment response. All biomarkers show high variability over time, but this limit can be minimized when using the biomarker within the appropriate clinical setting. All four biomarkers should be used, preferentially in combination, for asthma phenotyping and unrevealing the different Type-2 endotypes. For example, the major clinical applications of IgE are to predict the response to anti-IgE therapy and to determine its optimal dosage. The precise role of the choice of the right biologic treatment and response evaluation for personalized treatment in severe asthma is still to be fully elucidated. The level of strength of biomarkers in asthma clinical practice is summarized in Figure 2.

## Author contributions

GG and DB contributed to the conception and design of the work, drafting of the work, figures and tables elaboration, revision, interpretation, and critical analysis of the content. VC, FB, FR, SN, LB, EN, and GP contributed to design and drafting of the work and figures and tables elaboration. GC and EH contributed to the conception and design of the work, revision, interpretation, and critical analysis of the content. All authors contributed to manuscript revision, read, and approved the submitted version.

## Conflict of interest

The authors declare that the research was conducted in the absence of any commercial or financial relationships that could be construed as a potential conflict of interest.

## Publisher's note

All claims expressed in this article are solely those of the authors and do not necessarily represent those of their affiliated organizations, or those of the publisher, the editors and the reviewers. Any product that may be evaluated in this article, or claim that may be made by its manufacturer, is not guaranteed or endorsed by the publisher.
